# Exosomal miR-223 Contributes to Mesenchymal Stem Cell-Elicited Cardioprotection in Polymicrobial Sepsis

**DOI:** 10.1038/srep13721

**Published:** 2015-09-08

**Authors:** Xiaohong Wang, Haitao Gu, Dongze Qin, Liwang Yang, Wei Huang, Kobina Essandoh, Yigang Wang, Charles C. Caldwell, Tianqing Peng, Basilia Zingarelli, Guo-Chang Fan

**Affiliations:** 1 Department of Pharmacology and Cell Biophysics, University of Cincinnati College of Medicine, Cincinnati, OH, USA; 2 Department of Pathology and Laboratory Medicine , University of Cincinnati College of Medicine, Cincinnati, OH, USA; 3 Department of Surgery, University of Cincinnati College of Medicine, Cincinnati, OH, USA; 4 Shanxi Medical University, Taiyuan, China; 5 Shanxi University of Traditional Chinese Medicine, Taiyuan, China; 6 Critical Illness Research, Lawson Health Research Institute, Ontario, Canada N6A 4G5; 7 Division of Critical Care Medicine, Cincinnati Children’s Hospital Medical Center, Cincinnati, OH, USA

## Abstract

Mesenchymal stem cells (MSCs) have been shown to elicit cardio-protective effects in sepsis. However, the underlying mechanism remains obscure. While recent studies have indicated that miR-223 is highly enriched in MSC-derived exosomes, whether exosomal miR-223 contributes to MSC-mediated cardio-protection in sepsis is unknown. In this study, loss-of-function approach was utilized, and sepsis was induced by cecal ligation and puncture (CLP). We observed that injection of miR-223-KO MSCs at 1 h post-CLP did not confer protection against CLP-triggered cardiac dysfunction, apoptosis and inflammatory response. However, WT-MSCs were able to provide protection which was associated with exosome release. Next, treatment of CLP mice with exosomes released from miR-223-KO MSCs significantly exaggerated sepsis-induced injury. Conversely, WT-MSC-derived-exosomes displayed protective effects. Mechanistically, we identified that miR-223-KO exosomes contained higher levels of Sema3A and Stat3, two known targets of miR-223 (5p & 3p), than WT-exosomes. Accordingly, these exosomal proteins were transferred to cardiomyocytes, leading to increased inflammation and cell death. By contrast, WT-exosomes encased higher levels of miR-223, which could be delivered to cardiomyocytes, resulting in down-regulation of Sema3A and Stat3. These data for the first time indicate that exosomal miR-223 plays an essential role for MSC-induced cardio-protection in sepsis.

Sepsis is a systemic inflammation response to a local severe infection that can lead to multiple organ failure and ultimately, death^1^. Cardiac injury and dysfunction, commonly observed in septic patients, contribute substantially to the cardiovascular collapse, resulting in poor perfusion of blood into multiple tissues^2^. Therefore, strategies aimed to protect depressed hearts during sepsis would provide beneficial effects on mortality in this complex disease. Over the past years, mesenchymal stem cells (MSCs) originating from either bone marrow or adipose tissue have been consistently shown effective at reducing mortality and improving myocardial function in endotoxin-treated animals and preclinical models of polymicrobial sepsis induced by cecal ligation and puncture (CLP)^3^,^4^,^5^,^6^,^7^. The beneficial role of MSCs in these studies was believed to be primarily attributed to the interaction of MSCs with host macrophages in circulation and tissues, resulting in a reduced secretion of pro-inflammatory cytokines (i.e., TNF-α, IL-1β, and IL-6) from macrophages^3^,^4^,^5^,^6^,^7^. However, it remains unclear how MSCs interact with macrophages and other types of cells during sepsis. As a matter of fact, it has been documented that MSCs, when infused systemically in septic animal models, home mainly to the lung and the liver in a short time (5-10min after injection)^3^,^5^,^8^,^9^. Especially, these MSCs are not able to be detected in cardiac tissue^5^. Hence, MSC-induced cardiac benefits during sepsis may not be related to their local actions but their systemic effects. Nonetheless, the mechanisms underlying MSC-mediated cardio-protection in sepsis are still obscure.

Recently, exosomes have been widely reported to mediate local and systemic cell-to-cell communication^10^,^11^,^12^,^13^. They are nanometer-sized membrane vesicles (30–100 nm) released from numerous cell types upon fusion of multivesicular bodies (late endosomes) with the cell membrane. Numerous studies have demonstrated that exosomes can transfer a specific set of functional RNAs (miRNAs and mRNAs) and proteins into recipient cells through direct fusion of exosomes with the cell membrane or through active uptake, mediated by endocytosis^10^,^11^,^12^,^13^. Of interest, several recent studies have implicated exosomes as key effectors of MSC paracrine function and shown that exosomes released from MSCs were able to improve recovery in animal models of kidney failure, liver fibrosis, myocardial ischemia/reperfusion injury, hypoxia-induced pulmonary hypertension, and cerebral ischemia^14^,^15^,^16^,^17^,^18^. However, whether exosomes also contribute to MSC-induced cardio-protection against septic shock remains to be clarified.

Currently, it is well recognized that the functional significance of exosomes is dependent on the exosomal contents (miRNAs, mRNAs and proteins)^11^. In particular, miRNAs have been implicated as important exosomal components and largely decide the effects of exosomes on recipient cells^11^. For example, miR-146a is highly enriched in exosomes released from cardiosphere-derived cells and confers protection against myocardial infarction, whereas their capacity to protect stressed hearts is diminished by knockdown of exosomal miR-146a^19^. MiR-223 is the most highly expressed miRNA in both human peripheral blood mononuclear cells (PBMCs) and animal bone marrow-derived mesenchymal stem cells (MSCs)^20^,^21^. Significantly, miR-223 is highly encased in exosomes released from PBMCs and MSCs^20^,^21^. Numerous studies have indicated that miR-223 can negatively regulate the expression of many inflammatory genes (i.e., IL-6 and NLRP3)^21^. Importantly, our prior work also showed that loss of miR-223 aggravated myocardial depression and mortality in polymicrobial sepsis through up-regulation of Sema3A and Stat3, two known inflammation-related genes^22^. Therefore, we speculated that miR-223 may be critical for MSC-elicited action in sepsis. To this end, miR-223-KO MSCs were employed in septic mice induced by cecal ligation and puncture (CLP), and wild-type (WT) MSCs were used as controls. We further determined the effects of exosomes released from miR-223-KO MSCs on sepsis-induced inflammatory response, cardiac dysfunction and mortality. Finally, the possible underlying mechanisms were identified in this work. We believe that our study may provide a novel basis to the development of cell-free therapeutic approach for the treatment of sepsis.

## Results

### Administration of miR-223-KO MSCs does not improve animal survival and cardiac function in CLP-induced sepsis model

**Figure 1:**
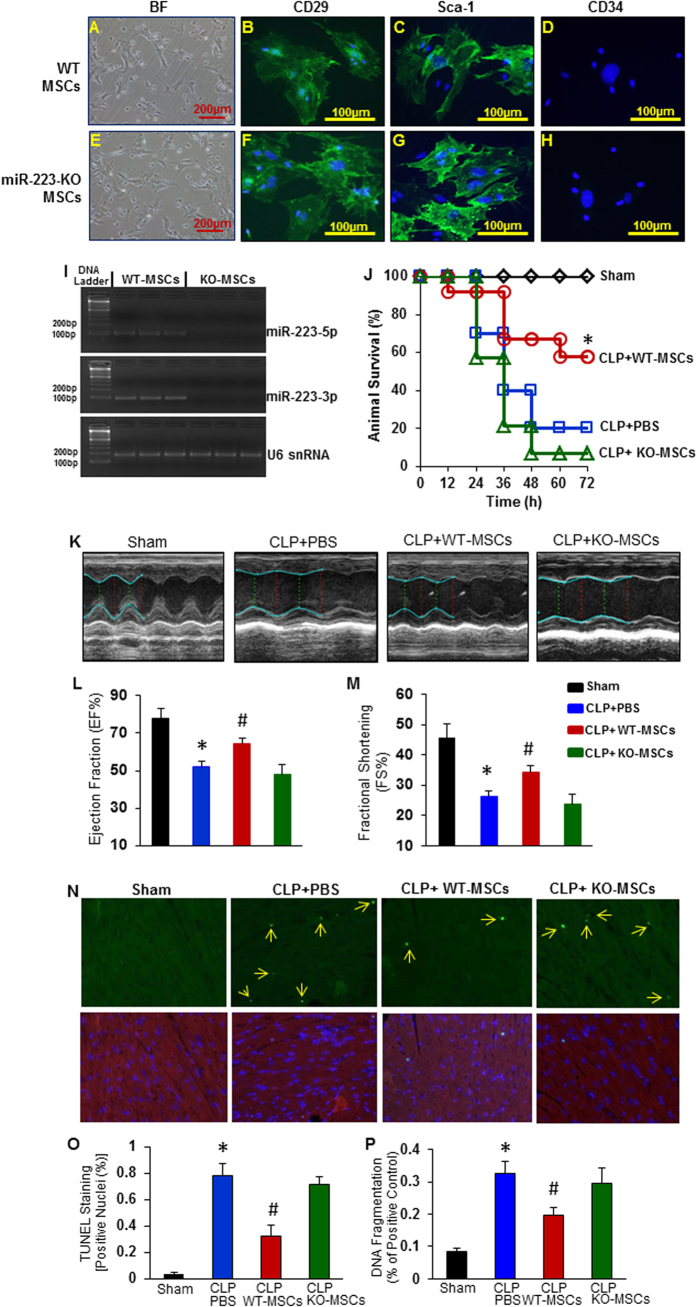
Absent of miR-223 impairs MSC-induced protection against sepsis-triggered cardiac injury. (**A**–**H**) Characterization of MSCs derived from bone marrow of WT mice (**A**–**D**) and miR-223 KO mice (**E**–**H**). (**I**) Total RNA was isolated from MSCs and performed RT-PCR analysis, and showed that miR-223-5p and -3p both are absent in KO-MSCs. U6 snRNA was used as internal control. (**J**) Injection of miR-223-KO MSCs (n = 14) at 1 h post-CLP did not improve animal survival, whereas the survival rate was significantly improved in WT-MSC-treated mice (n = 12), compared with PBS controls (n = 10, *p < 0.05). (**K**–**M**) CLP-induced cardiac depression, measured by echocardiography (**K**), was significantly attenuated in WT-MSC-treated mice, but not in KO-MSC-treated mice. *p < 0.05, vs. sham group; #*p* < 0.05, vs. CLP+PBS control group; n = 6 for sham group, n = 10 for CLP + PBS, n = 12 for WT- and KO-MSC-treated groups. (**N**) Representative TUNEL staining for detection of apoptotic cardiomyocytes (green dots indicated by arrows, upper row). Cardiomyocytes were stained with α-actin (Red) and nuclei were stained with DAPI (blue), which were merged together, indicated in the lower row. (**O**) Quantitative results of TUNEL staining, which was further confirmed by ELISA measurement of histone-associated DNA fragmentation (**P**). (n = 5 hearts, *p < 0.05, *vs*. shams; #*p* < 0.05 *vs.* CLP+PBS control group).

To address whether miR-223 contributes to MSC-induced protection in sepsis, we harvested MSCs from bone marrow of female miR-223 KO (miR-223^−/−^) mice. MSCs derived from female wild-type (WT) bone marrow were used as controls. MiR-223-KO MSCs were phenotypically similar to WTs, and expressed mesenchymal markers (i.e. CD29 and Sca-1), but not hematopoietic markers (i.e. CD34) (Fig. 1A–H). We also validated that the expression of both strands (miR-223-5p and miR-223-3p) was deficient in MSCs collected from miR-223-KO mice (Fig. 1I). To understand the functional significance of miR-223 in MSC-mediated protection against sepsis injury, miR-223-KO MSCs and controls were administered intravenously to male WT mice at 1 h post-CLP surgery. We observed that survival rate was significantly improved in CLP-mice treated with WT-MSCs, as evidenced by 58% of the mice (n = 12) survived until the end of day 3, when only 20% of PBS-treated mice survived (n = 10) (Fig. 1J). These results are consistent with previous reports^3^. However, CLP-mice treated with miR-223-null MSCs (n = 14) did not exhibit an improved survival, compared to PBS-treated controls (Fig. 1J). This suggests that miR-223 may be essential for MSC-elicited beneficial effects on survival during polymicrobial sepsis.

We next determined whether miR-223 affects MSC-induced cardio-protection in sepsis. Cardiac function was measured by echocardiography in CLP mice treated with miR-223-KO MSCs or controls. We observed that hearts of CLP-mice treated with WT-MSCs had improved values of left ventricular ejection fraction (EF %) and fractional shortening (FS %), compared with the vehicle PBS group at 12 h post-CLP (Fig. 1K–M, *p *< 0.05). By contrast, no difference in either EF% or FS% was identified between the miR-223-KO MSC- and the vehicle-treated groups (Fig. 1K–M).

Numerous studies have indicated that sepsis-induced myocardial dysfunction may be directly ascribed to cardiomyocyte damage^23^,^24^,^25^. As a matter of fact, few apoptotic cadiomyocytes were detected by TUNEL staining in sham-operated mice (Fig. 1N,O). However, extensive apoptotic nuclei (∼8/1000 nuclei) were observed in the myocardium of vehicle-treated mice at 12 h post-CLP surgery (Fig. 1N,O). Importantly, infusion of WT-MSCs, but not miR-223-KO MSCs, into CLP-mice reduced cardiomyocyte apoptosis to a greater degree (∼3/1000 nuclei), compared with vehicle-treated hearts (Fig. 1N,O). These results were further validated by myocardial DNA fragmentation assays, using an ELISA method (Fig. 1P). Collectively, these data suggest that MSC-mediated cardio-protection in sepsis may be dependent on the expression of endogenous miR-223.

### Loss of miR-223 in MSCs impairs the inhibitory effects on systemic inflammation elicited by WT-MSCs.

**Figure 2:**
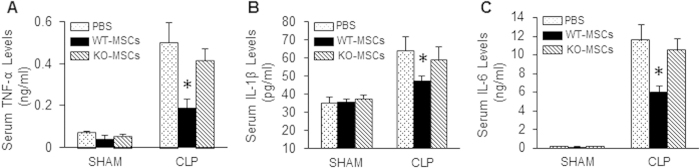
Loss of miR-223 in MSCs negates MSC-induced inhibitory effects on CLP-triggered systemic inflammatory response. Injection of WT-MSCs into mice at 1h post-CLP significantly reduced the circulating levels of inflammatory cytokines: (**A**) TNF-α, (**B**) IL-1β, and (**C**) IL-6, whereas they were not reduced in KO-MSC-treated mice, compared with PBS-treated samples. *p < 0.05, n = 4 for shams, n = 6–8 for CLP mice.

It is well accepted that sepsis-triggered cardiac injury (i.e., apoptosis and dysfunction) may be indirectly linked to the presence of cardio-suppressive cytokines in serum^1^,^2^. Given that MSCs can exert a strong inhibitory effect on sepsis-induced systemic inflammation^3^,^4^,^5^,^6^,^7^, we next measured the levels of pro-inflammatory cytokines (TNF-α, IL-1β, and IL-6) in the sera collected from MSC-treated mice at 12 h post-CLP. As shown in Fig. 2A–C, under sham conditions, injection of either WT-MSCs or KO-MSCs into mice did not change the circulating levels of inflammatory cytokines (TNF-α, IL-1β, and IL-6), compared with PBS controls. However, treatment of CLP-mice with WT-MSCs significantly reduced serum levels of TNF-α, IL-1β, and IL-6, compared to PBS-treated samples (Fig. 2A–C), which is consistent with previous reports^3^,^4^,^5^. By contrast, these cytokine levels only slightly decreased, but not significant, in the blood of miR-223-KO/MSC-treated CLP-mice, comparable to PBS-samples (Fig. 2A–C). Put together, these data suggest that the immune-inhibitory property of MSCs is associated with miR-223.

### WT-MSCs suppress LPS-induced cytokine release from macrophages to a greater degree than miR-223-KO MSCs

**Figure 3:**
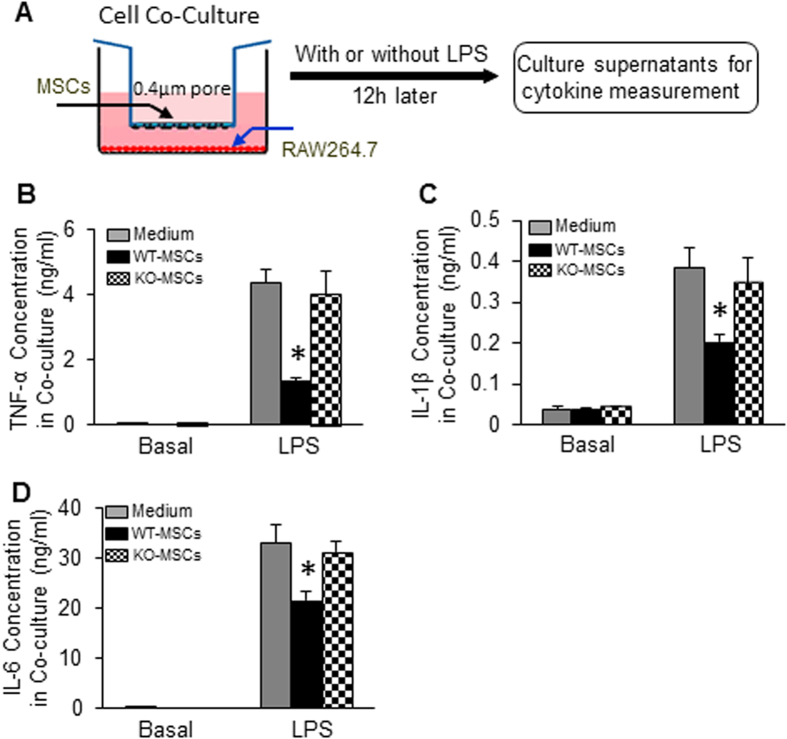
MiR-223 is critical for MSC-mediated inhibitory effects on the cytokine production in macrophages upon LPS challenge. (**A**) A diagram of cell co-culture system in which macrophages (RAW264.7 cells) were cultured in the lower chamber and MSCs were cultured in the upper chamber of a 12-well insert. (**B**–**D**) Inhibition of LPS-triggered TNF-α (**B**), IL-1β (**C**), and IL-6 (**D**) production was more remarkable in macrophages co-cultured with WT-MSCs than those co-cultured with KO-MSCs. (n = 3 wells, *p < 0.05, *vs.* Medium controls and KO-MSC samples). Similar results were observed in two additional, independent experiments.

It is well appreciated that the large amounts of pro-inflammatory cytokines triggered by bacterial infection are mostly released from macrophages^1^. Importantly, macrophages, as autocrine and paracrine signaling mediators, could initiate further inflammatory responses in both neighboring macrophages and other types of cells^1^. To determine whether MSCs could directly suppress sepsis-induced inflammatory cytokine release from macrophages, we employed a well-controlled *in vitro* system of cell co-culture in which mouse MSCs were cultured in the upper chamber and RAW264.7 macrophages were seeded in the lower chamber (Fig. 3A), followed by treatment with bacterial endotoxin, LPS. We observed that LPS remarkably stimulated macrophages to release cytokines (TNF-α, IL-1β, and IL-6) (Fig. 3B–D). However, when macrophages were co-cultured with WT-MSCs, LPS-caused production of TNF-α, IL-1β, and IL-6 was dramatically inhibited (Fig. 3B–D). Notably, miR-223-KO MSCs did not significantly attenuate the secretion of these pro-inflammatory factors in macrophages upon LPS challenge (Fig. 3B–D). These results suggest: 1) immune-regulatory mechanisms of MSCs in sepsis may take effect through paracrine factors rather than direct cell contact; and 2) endogenous miR-223 may be critical, at least in part, for MSC-elicited immuno-suppression.

### Blockade of exosome secretion negates the inhibitory effects of WT-MSCs on LPS-triggered cytokine production in macrophages and attenuates protective effects of WT-MSCs on LPS-induced cardiomyocyte death

**Figure 4:**
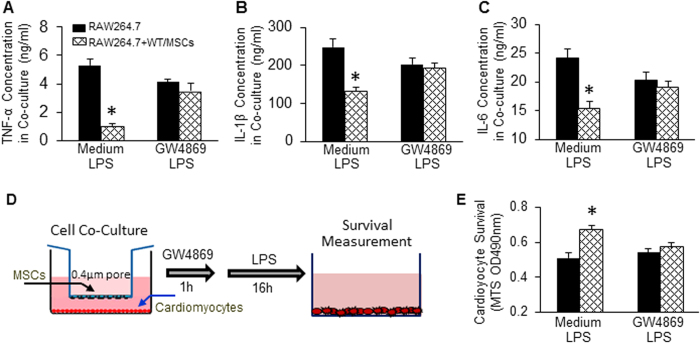
Blockade of exosome release negates the inhibitory effects of WT-MSCs on LPS-triggered inflammatory cytokine production in macrophages and LPS-induced cardiomyocyte death. WT-MSCs significantly suppressed the production of TNF-α (**A**), IL-1β (**B**), and IL-6 (**C**) in co-cultured RAW264.7 cells upon LPS challenge. Such inhibitory effects were offset by addition of GW4869 (20 μM). n = 3 wells, *p < 0.05, *vs.* RAW264.7 cultured alone. Similar results were observed in three additional, independent experiments. (**D**) A diagram of cell co-culture system in which cardiomyocytes were cultured in the lower chamber of a 12-well plate pre-coated with laminin (10 μg/ml) and MSCs were cultured in the upper chamber of a 12-well insert. (**E**) LPS exposure significantly decreased cardiomyocyte survival, whereas it is greatly improved by co-culturing with WT-MSCs. n = 3 wells, *p < 0.05, *vs.* cardiomyocytes cultured alone. Addition of GW4869 (20 μM) offset WT-MSC-elicited protective effects on LPS-triggered myocyte death. Similar results were observed in two additional, independent experiments.

In recent years, exosomes have emerged as one of the most important players for both local and long-distance cell-cell communication^10^,^11^. To examine whether WT-MSC-induced effects are associated with their released exosomes, we pre-treated WT-MSCs with GW4869 (20 μM), an inhibitor of exosome biogenesis and release^13^,^19^,^26^,^27^, then co-cultured with macrophages, followed by LPS stimulation for 12 h. In this study, we observed that the TNF-α concentration was reduced by 80% in co-culture supernatants, compared with macrophages cultured alone (p* *< 0.05) (Fig. 4A). However, the concentration of TNF-α in co-culture supernatants was similar between groups upon addition of GW4869 (Fig. 4A). Likewise, the secretion of IL-1β and IL-6 from macrophages was remarkably inhibited by WT-MSCs, but such a suppression was able to escape from pre-treatment with GW4869 (Fig. 4B,C). These results indicate that blockade of exosome generation with GW4869 impairs MSC-elicited anti-inflammatory effects.

To further investigate whether MSC-released exosomes contribute to MSC-mediated cardiomyocyte survival in sepsis, we co-cultured adult rat cardiomyocytes with MSCs, followed by pre-treatment with GW4689 for 1 h and then addition of LPS (1 μg/ml) for 16 h (Fig. 4D). Cardiomyocyte survival was measured by MTS incorporation (at that time, upper chamber containing MSCs was removed). We observed that cardiomyocyte survival was significantly improved when co-cultured with WT-MSCs (Fig. 4E). By contrast, upon addition of GW4689 to the co-cultures, such protective effects on myocyte survival were lost (Fig. 4E). Hence, MSC-induced beneficial effects on cardiomyocytes during sepsis may be through exosomes.

### WT-MSC exosomes suppress LPS-induced pro-inflammatory cytokine release from macrophages and protect cardiomyocytes against LPS injury, whereas miR-223-KO exosomes exhibits opposite effects

**Figure 5:**
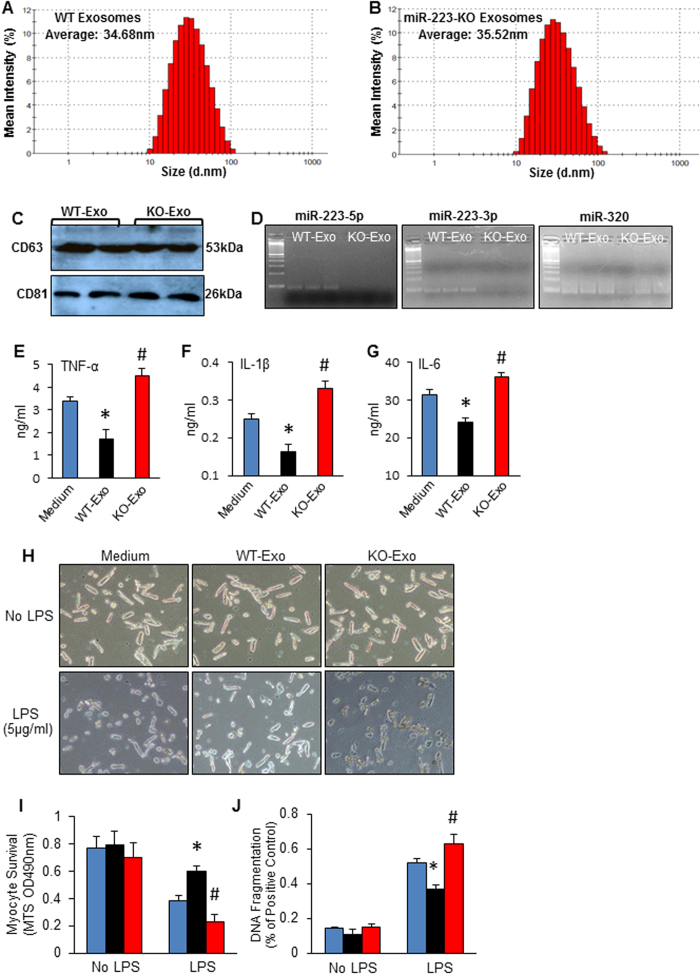
Characterizations of exosomes derived from MSCs and their functional roles in macrophages and cardiomyocytes upon LPS challenge. (**A**,**B**) The size of exosomes derived from (**A**) WT-MSCs and (**B**) miR-223 KO-MSCs, measured using a Zetasizer Nano ZS instrument. (**C**) Protein levels of CD63 and CD81 were similarly encased in WT-exosomes and KO-exosomes. Figure represents truncated western blot images for simplicity. Whole membrane images are shown in Supplementary Figure S2. (**D**) Both strands of miR-223 were included in WT-exosomes and null in KO-exosomes, 100 bp-DNA lander was used as a gel loading marker. MiR-320 was used as an internal control for RT-PCR. n = 3 independent experiments for A–D. (**E**–**G**) Addition of WT-exosomes (20 μg/ml) to cultured RAW264.7 cells significantly inhibited LPS-triggered secretion of TNF-α (**E**), IL-1β (**F**), and IL-6 (**G**). Remarkably, KO-exosomes (20 μg/ml) promoted RAW264.7 cell secretion of TNF-α (**E**), IL-1β (**F**), and IL-6 (**G**) upon LPS challenge (100 ng/ml). n = 3 wells for each group; *p < 0.05, *vs.* medium controls; #*p* < 0.05, *vs.* medium controls. Similar results were observed in other two additional, independent experiments. (**H**–**J**) LPS-induced cardiomyocyte death/apoptosis was significantly mitigated by treatment with WT-exosomes (20 μg/ml) and remarkably promoted by addition of KO-exosomes (20 μg/ml). Representative images of cardiomyocytes in the absent and present of LPS plus WT-exosomes or KO-exosomes were shown in (H). Survival rate was determined by MTS incorporation (**I**), and cardiomyocyte apoptosis (DNA fragmentation) was determined using an ELISA kit (**J**). n = 3 wells for each group; *p < 0.05, *vs.* medium controls; #*p* < 0.05, *vs.* medium controls. Similar results were observed in other three additional, independent experiments.

To determine whether MSC-derived exosomes have the capacity of anti-inflammation and promoting cell survival, we purified exosomes from the culture supernatants of MSCs by serial differential centrifugation plus ultracentrifugation^27^. Using a particle size analyzer, we observed that the size of exosomes released from WT-MSCs (WT-Exo) ranged between 10–100 nm with an average of 34.68 nm, which was similar to that of miR-223-KO MSC-derived exosomes (KO-Exo: 35.52 nm) (Fig. 5A,B). In addition, both WT-Exo and KO-Exo contained similar levels of CD63 and CD81 (Fig. 5C), two widely recognized molecular markers for exosomes^10^,^11^,^12^,^13^. Using RT-PCR assays, we also confirmed that both strands of miR-223 were encased in WT-exosomes, which were absent in KO-exosomes (Fig. 5D). Notably, we utilized sucrose gradient ultracentrifugation to further purify exosomes from culture supernatants of MSCs, as described previously^28^, and also confirmed that miR-223 was included in exosomes derived WT-MSCs rather than KO-MSCs (Supplemental Fig. S1). To determine the effects of these MSC-derived exosomes on macrophage inflammatory response, we added either WT-exosomes or KO-exosomes to cultured RAW264.7 cells. One hour later, LPS (100 ng/ml) was added to these cultures for 12 h, and then supernatants were collected for cytokine measurements. The results of ELISA analysis revealed that addition of WT-exosomes significantly inhibited the secretion of TNF-α, IL-1β, and IL-6 from macrophages upon LPS stimulation, evidenced by lower levels of TNF-α, IL-1β, and IL-6 in culture supernatants of WT-exosome-treated macrophages than those of medium-treated controls (Fig. 5E–G). By contrast, LPS-stimulated production of TNF-α, IL-1β, and IL-6 was remarkably increased in miR-223-KO exosome-treated macrophages, compared with medium-treated samples (Fig. 5E–G).

To explore whether MSC-derived exosomes could affect LPS-induced cardiomyocyte injury, we added either WT-exosomes or KO-exosomes to cultured cardiomyocytes, followed by LPS challenge. Remarkably, miR-223-containing exosomes (WT-exosomes) protected cardiomyocytes against LPS-induced cell death/apoptosis, evidenced by higher degree of cell survival and lower degree of cellular DNA fragmentation in WT-exosome-treated cardiomyocytes than those exposed to medium controls (Fig. 5H–J). Oppositely, miR-223-null exosomes greatly augmented LPS-triggered cell death/apoptosis, compared with controls (Fig. 5H–J). Taken together, these data suggest that absence of miR-223 is not able to change the size of exosomes released from MSCs, but could impair the beneficial effects of MSC-derived exosomes on inhibition of macrophage inflammatory response and cardiomycoyte death during sepsis.

### Injection of WT-exosomes attenuates CLP-induced systemic inflammatory response, whereas treatment with miR-223-KO exosomes displays opposite effects

**Figure 6:**
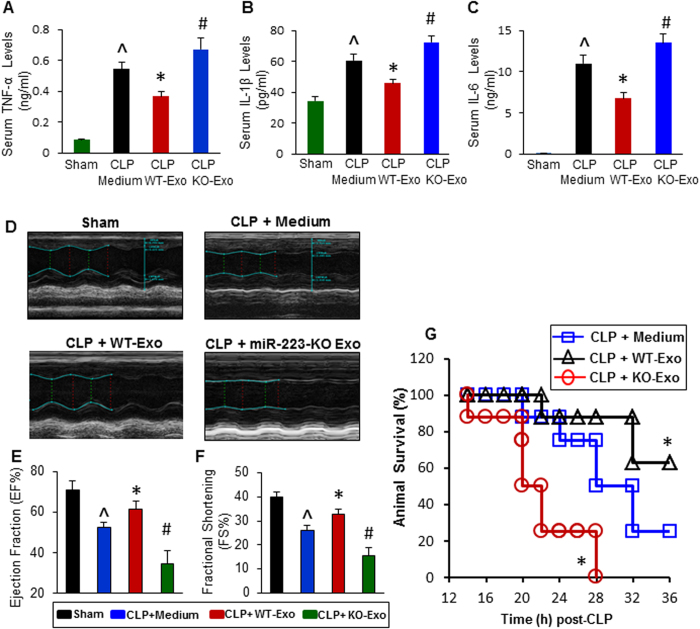
The effects of WT-exosomes and miR-223-KO exosomes on CLP-induced inflammatory response, cardiac dysfunction and animal mortality. (**A**–**C**) CLP-mice treated with WT-exosomes (n = 11) showed lower levels of serum TNF-α (**A**), IL-1β (**B**), and IL-6 (**C**), whereas CLP-mice injected with KO-exosomes (n = 11) exhibited higher levels of circulating TNF-α (**A**), IL-1β (**B**), and IL-6 (**C**), compared with those treated with incomplete DMEM medium (n = 10) (^*p* < 0.05 *vs*. shams; *p < 0.05 *vs*. CLP + medium; #*p* < 0.05 *vs*. CLP + medium). (**D**) Results of echocardiography measurement showed that values of the left ventricular ejection fraction (EF%, E) and the fractional shortening (FS%, F) were significantly decreased in CLP mice injected with incomplete DMEM medium (n = 10), compared with shams (n = 8). Remarkably, the reduction of EF% and FS% was attenuated in WT-exosome-treated CLP mice (n = 11); whereas it was aggravated in CLP mice administrated with miR-223-KO exosomes (n = 11) (^*p* < 0.05 *vs*. shams; *p < 0.05 *vs*. CLP + medium; #*p* < 0.05 *vs*. CLP + medium). (**G**) The survival of CLP-mice was significantly improved by WT-exosome treatment, whereas it was worse by miR-223-KO exosome injection (n = 8, *p < 0.05 *vs*. CLP + medium).

Next, we asked whether MSC-derived exosomes had immune-suppressive effects, similar to MSCs *in vivo*, and if so, whether miR-223 is required for such effects. To address these issues, we infused WT-exosomes, KO-exosomes, or incomplete culture medium into mice *via* the tail vein at 1 h post-CLP surgery. 12 h later, sera were collected from those mice for cytokine assays. As shown in Fig. 6A–C, the serum levels of TNF-α, IL-1β, and IL-6 were greatly reduced in WT-exosome-treated CLP-mice, whereas the serum levels of these pro-inflammatory factors were significantly increased in KO-exosome-injected septic mice, compared with medium-treated control samples (n = 8–11). These data suggest that: 1) MSC-derived exosomes can be used in place of intact stem cells to inhibit sepsis-induced inflammatory response; and 2) MSC-exosome-induced anti-inflammatory effects are dependent on miR-223, at least in part.

### Exosomes derived from WT-MSCs attenuate cardiac dysfunction and improve animal survival in polymicrobial sepsis, whereas exosomes released from miR-223-KO MSCs yield opposite effects

To further determine whether myocardial function is altered in those septic mice treated with exosomes, we measured contractile function with echocardiography at 12 h post-CLP surgery (Fig. 6D). Similar to the animals treated with WT-MSC (Fig. 1K–M), exosomes derived from WT-MSCs protected against CLP-induced heart failure, as evidenced by an improvement of both left ventricular ejection fraction (EF %) and fractional shortening (FS %), compared with incomplete culture medium-treated CLP mice (Fig. 6E,F). However, unlike the CLP mice treated with miR-223-KO MSCs (Fig. 1K–M), exosomes derived from miR-223-KO MSCs aggravated cardiac dysfunction in septic mice, comparable to those CLP mice treated with control medium ((Fig. 6E,F, n = 8–11). The dissimilarity in functional consequence may be ascribed to other beneficial factors released from miR-223-null MSCs in addition to these detrimental exosomes. In parallel, another set of experiment for animal survival assays revealed that treatment of CLP mice with WT-exosomes significantly improved animal survival (5 out 8 survived at 36 h post-CLP) (Fig. 6G), compared to medium-injected group. By contrast, all mice treated with miR-223-KO exosomes died at 28 h post-CLP (Fig. 6G). Taken together, these observations suggest that MSC-released exosomes may have therapeutic effects on sepsis-induced heart failure and mortality, which is associated with exosomal miR-223.

### WT-exosomes transfer miR-223, whereas KO-exosomes transport Stat3 and Sema3A proteins, to the myocardium *in vivo*

**Figure 7:**
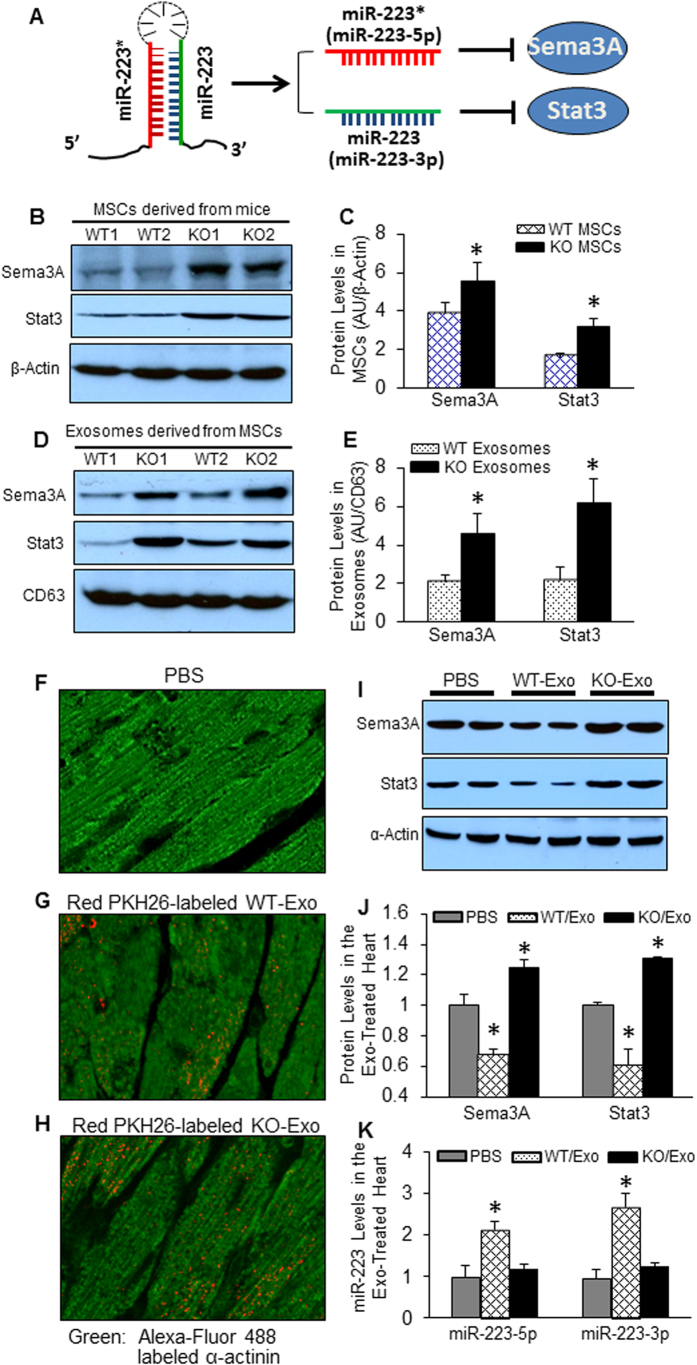
miR-223-KO exosomes can deliver Sema3A and Stat3, whereas WT-exosomes can deliver miR-223 to the myocardium *in vivo*. (**A**) A diagram shows that both strands of pre-miR-223 can be processed to be mature miRNA and target Sema3A and Stat3, respectively. (**B**,**C**) Sema3A and Stat3 both were up-regulated in miR-223-KO MSCs (n = 4, *p < 0.05 *vs*. WTs). (**D**,**E**) Sema3A and Stat3 both proteins were highly enriched in exosomes released from miR-223-KO MSCs (n = 4, *p < 0.05 *vs*. WTs). (**F**–**H**) Red dye PKH26-labeled WT- and KO-exosomes both were detected in the myocardium after *i.v.* injection. Cardiomyocytes were stained with Alexa-Fluor 488 labeled α–actinin antibody. **(I**,**J**) Higher levels of Sema3A and Stat3 encased in miR-223-KO exosomes were effectively transported to the heart, whereas WT-exosome-treated hearts displayed lower levels of Sema3A and Stat3, compared to PBS-injected control hearts, respectively (n = 4, *p < 0.05 *vs*. PBS-treated samples). α-actin was used as a loading control. AU: arbitrary unit. The gel had been run under the same experimental conditions. The full-length blots are shown in Supplementary Figure S3. (**K**) The levels of miR-223-5p and -3p were significantly increased in WT-exosome-treated myocardium, whereas they were not altered in KO-exosome-treated hearts. U6 snRNA was used as an internal control for qRT-PCR analysis (n = 4, *p < 0.05 *vs*. PBS controls).

We next tried to figure out the possible mechanisms underlying the beneficial effects of WT-exosomes and detrimental effects of miR-223-KO-exosomes in sepsis. It has been shown that: 1) miR-223 is highly enriched in not only bone marrow stem cells but also their released exosomes (Fig. 5D and Ref. 20); 2) miR-223-5p and -3p could negatively regulate the expression of Sema3A andStat3, respectively^22^ (Fig. 7A); and 3) Sema3A and Stat3 both contribute to inflammatory response^29^,^30^,^31^,^32^. We therefore speculated that the presence or absence of miR-223 in MSCs might affect cellular levels of Sema3A and Stat3, which were consequently incorporated into exosomes at different concentrations. To test this hypothesis, we firstly determined the expression levels of Sema3A and Stat3, two major inflammatory targets of miR-223, in MSCs collected from miR-223-KO mice and WT (miR-223^+/+^) mice. The results of Western-blotting revealed that the levels of Sema3A and Stat3 were increased in miR-223-KO MSCs by 1.4-fold and 1.9-fold, respectively, compared with WT-MSCs (Fig. 7B,C, *p *< 0.05). Of importance, we observed that exosomes released from miR-223-KO MSCs contained higher levels of Sema3A (2.1-fold) and Stat3 (2.8-fold) than WT-exosomes (Fig. 7D,E, *p *< 0.05). These data implicate that ablation of miR-223 in stem cells is able to reprogram the protein contents of exosomes.

To determine whether exosomes could be taken up by cardiac cells *in vivo*, we labeled exosomes with the red fluorescent membrane dye PKH26 and injected to mice *via* the tail vein. Eight hours later, the majority of cardiomyocytes acquired the red dye-labeled exosomes and importantly, both WT-exosomes and KO-exosomes were distributed similarly in the cytoplasm of cardiomyocytes (Fig. 7F–H). These results are consistent with previous reports showing that MSC-derived exosomes could be detected in multiple tissues/organs (i.e. liver, kidney and heart) after *i.v.* injection^33^,^34^,^35^. Accordingly, hearts collected from miR-223-KO-exosome-treated mice displayed higher levels of Sema3A and Stat3 than PBS-injected samples (Fig. 7I,J). By contrast, WT-exosome-treated hearts revealed lower levels of Sema3A and Stat3 than PBS-controls (Fig. 7I,J). This might be attributed to WT-exosome-mediated delivery of miR-223 to the heart. As a matter of fact, RT-PCR results showed that the levels of miR-223-5p and -3p were significantly increased in WT-exosome-treated hearts, compared with PBS controls (Fig. 7K). By contrast, KO-exosome-treated hearts did not show the increased levels of miR-223 (5p and 3p) (Fig. 7K). Put together, these results indicate that WT-exosomes could deliver miR-223 to the myocardium and consequently, inhibit the expression of Sema3A and Stat3, two *bona fide* targets of miR-223; whereas miR-223-KO exosomes could deliver Sema3A and Stat3 proteins to the myocardium.

## Discussion

**Figure 8:**
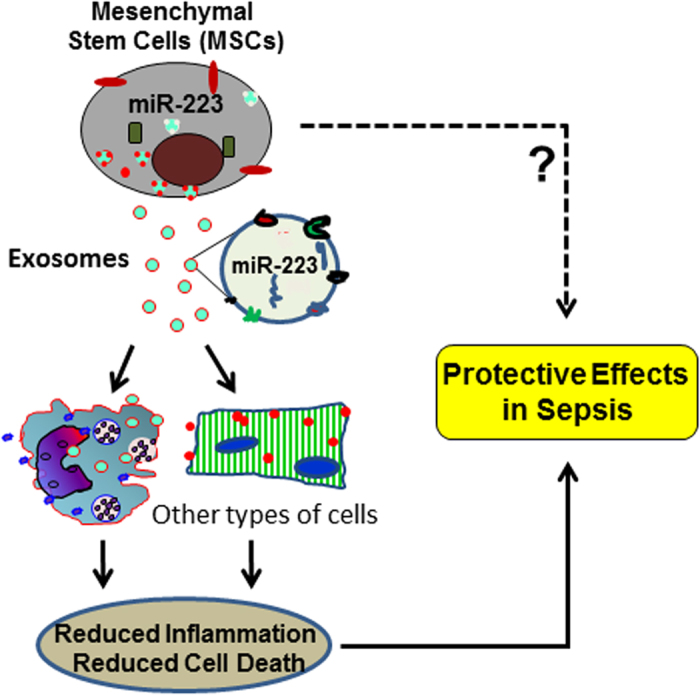
A work model elucidating that miR-223 contributes to MSC-elicited protective effects against sepsis through the exosome-mediated transfer of miR-223 to other types of cells (i.e. macrophages and cardiomyocytes), leading to attenuation of inflammatory response and inhibition of cell death in recipient cells.

In this study, we center on elucidating the mechanisms underlying MSC-mediated cardio-protection in sepsis. Major findings of the present study include: 1) miR-223-null MSCs did not improve myocardial function and survival rate when they were injected into septic mice; 2) blockade of exosome generation *in vitro* with GW4869 impaired MSC-induced suppression of inflammation in macrophages and offset MSC-elicited protective effects in cardiomyocytes upon LPS challenge; 3) miR-223-absent exosomes derived from KO-MSCs aggravated sepsis-induced heart failure, inflammatory response, and mortality through the exosomal transfer of Sema3A and Stat3 to the myocardium; and 4) miR-223-present exosomes derived from WT-MSCs attenuated sepsis-trigged myocardial depression through the exosomal transfer of miR-223 to recipient cells and consequently, down-regulated the expression of Sema3A and Stat3, leading to the reduction of inflammatory response and cell death (Fig. 8). Our findings for the first time suggest that MSC-induced protective effects on sepsis may be largely dependent on exosomal miR-223.

Nemeth *et al.*^3^ previously reported that MSC-mediated therapeutic effects on sepsis are ascribed to a contact-dependent interaction between MSCs and macrophages. However, this interpretation may not fit well with recent findings that, at 10 min after intravenous injection, 2–3% of initially injected MSCs were remained in the circulation, <1% of cells were detected in the lung, the liver and the spleen, and null in the heart^5^,^9^. Indeed, several recent studies have suggested that beneficial effects of MSCs on diseases are probably not primarily dependent on local mechanisms but through the secretion of paracrine factors^9^,^15^,^36^. Hence, our study timely provides the first evidence showing that MSCs are able to reprogram macrophages and cardiomyocytes during sepsis *via* the exosome-mediated transfer of miR-223. It is important to mention here that MSCs may be as “a manufactory” in which exosomes are continually generated.

MiR-223 was initially characterized in the hematopoietic system and was believed to be specifically expressed in the myeloid compartment^37^. Subsequently, it was found to be detectable in multiple tissues (i.e. heart, lung, kidney, liver, and spleen)^38^. Increasing evidence points to miR-223 as an important factor in the regulation of inflammatory response^21^,^22^,^39^,^40^,^41^. For example, Dorhoi *et al.* recently showed that miR-223 controls susceptibility to tuberculosis by regulating lung neutrophil recruitment^39^. Haneklaus *et al.* revealed that overexpression of miR-223 in THP-1 cells (a human acute monocytic leukemia cell line) prevents accumulation of NLRP3 protein and inhibits IL-1β production from the inflammasome^40^. Importantly, the miR-223 expression could be down-regulated by LPS in macrophages, leading to an increased secretion of IL-6 and IL-1β^29^. Recent clinical studies further showed that serum exosomal miR-223 levels were significantly reduced in non-survival septic patients, compared with survivals^42^. Our prior work also demonstrated that miR-223 was the most decreased miRNA in heart tissues collected from severe septic mice, and miR-223-KO mice were sensitive to sepsis-induced injury^22^. Therefore, all the observations consistently implicate alteration of miR-223 as a critical factor for the pathogenesis of inflammation-related disease, especially during the process of septic shock. In the present study, we provide novel evidence showing that miR-223 may be required for MSC-elicited inhibition of inflammatory response in sepsis. It is worthy to note here that the miR-223 gene is located at X-chromosome^37^. This means that female cells contain double levels of miR-223, compared to male cells. Hence, our data may partially interpret previous observations that female MSCs are superior to males in preserving myocardial function following endotoxemia^43^.

As for how miR-223 contributes to MSC-induced protective effects, it could be mediated by exosomes, a group of nano-vesicles actively released from MSCs. Currently, it is well recognized that exosomes containing miRNAs/proteins are key effectors of the MSC paracrine mechanism^11^. Numerous studies have indicated that exosomes could deliver their contents to different types of cells and importantly, donor exosomal miRNAs are functional in recipient cells^11^,^13^,^33^,^34^. Hence, through exosome-mediated transfer of highly enriched miRNAs, MSCs can “educate” recipient cells and influence their behavior. In this study, we showed that WT-MSCs could secret miR-223-enriched exosomes, which were subsequently taken up by recipient cells (i.e., macrophages and cardiomyocytes). As a result, the miR-223 targets (i.e., Sema3A and Stat3) were down-regulated, leading to the inhibition of inflammatory response in macrophages and attenuation of cardiomyocyte death during sepsis (Fig. 8). On the contrary, exosomes released from miR-223-absent MSCs contain higher levels of Sema3A and Stat3, which might be delivered to macrophages and cardiomyocytes, leading to promotion of macrophage inflammation and cardiomyocyte death in sepsis. It is important to note here, our results presented in this study showed that the effects of miR-223-null MSCs and their released exosomes on the inflammatory response were different. It may be easy to interpret that miR-223-null stem cells may not only secret “harmful” exosomes but also release some beneficial factors. As such, unlike the purified exosomes displaying promotion of sepsis-triggered inflammation, miR-223-null MSCs did not exaggerate sepsis-induced injury. We selected Sema3A and Stat3 for this study, because both are consistently validated to be *bona fide* targets of miR-223 in macrophages and cardiomyocytes^22^,^29^. Importantly, Sema3A has been shown to initiate sepsis-triggered cytokine storm by interacting with Plexin-A4 and Toll-like receptors (TLRs)^30^; Stat3 is well known to be a key upstream activator of inflammatory signaling pathways during sepsis^31^,^32^. Certainly, other miRNAs/proteins included in MSC-exosomes may also contribute to MSC-induced effects, which we could not exclude in this study. Nonetheless, we believe that our study may suggest that therapeutic effects of MSCs on sepsis are, at least in part, dependent on the axis of miR-223-Sema3A/Stat3 mediated by exosomes.

There are several limitations in the present study. Firstly, given that GW4869 is an inhibitor of neutral sphingomyelinase (nSMase)^26^ and nSMase is highly expressed in all tissues^44^, additional effects of GW4869 beyond inhibition of exosome secretion cannot be excluded. Secondly, our study did not examine the effects of MSCs and their released exosomes on other organ damage but cardiomyopathy triggered by sepsis. Accordingly, it is impossible to exclude the *in vivo* effects of exosomes on other cell types (i.e. macrophages and endothelial cells) in addition to cardiomyocytes. Such analysis may fall outside the scope and intent of this report. Nonetheless, the data present in this study raise a novel hypothesis that miR-223-containing exosomes derived from MSCs may represent a new effective therapeutic approach for the treatment of sepsis-induced multiple organ failure. First, exosomes, as cell-free membrane-vesicles, have many potential advantages over MSCs, such as less toxicity and a minimal immune response, relatively stable in the circulation, and convenient to handle than fully intact stem cells^11^,^45^. Second, exosome-mediated transfer of miRNA/proteins is not tissue-specific^11^. As a matter of fact, recent studies have shown that MSC-exosomes injected into mice *via* the tail vein can be detectable in the heart, the kidney, the lung, the liver, and the brain, leading to protection from ischemia/reperfusion-induced cardiac injury, chronic kidney disease, liver fibrosis, endotoxin-triggered acute lung injury and stroke^14^,^15^,^16^,^17^,^18^. Most importantly, diverse targets of miR-223 (5p and 3p) appear to function in concert to regulate inflammatory response during sepsis. For example, in addition to Stat3, miR-223-3p (previously referred to as miR-223) can negatively regulate the expression of numerous inflammation-related genes, such as Mef2C, PKnox1, CXCL2, CCL3, IL-6, and NLRP3^37^,^38^,^39^,^40^,^41^. Likewise, apart from Sema3A, miR-223-5p (also named as miR-223*) is predicted to target TRAF family member-associated NF-kappa B activator (TANK), tumor necrosis factor receptor superfamily member 21 [TNFRSF21, also known as death receptor 6 (DR6)], and interferon regulatory factor 4 (IRF4), etc (see database available at www. mirdb.org). Therefore, MSC-induced anti-inflammatory effects may be largely through exosomal transfer of miR-223 to multiple tissues/organs during sepsis.

In summary, our findings presented here for the first time demonstrate that: 1) cardio-protective effects of MSCs in sepsis are associated with exosomal miR-223; and 2) ablation of miR-223 in MSCs can reprogram protein contents of exosomes, which yields detrimental effects on recipient cells. Our study suggests that miR-223-bearing exosomes may have a great therapeutic potential for the treatment of sepsis.

## Material and Methods

### Animals

Mating pairs of miR-223 knockout mice on the background of C57BL/6 (male: miR-223^−/y^ and female: miR-223^−/−^) and wild-type C57BL/6 mice were purchased from Jackson Laboratory (Indianapolis, IN). Mice were maintained and bred in the Division of Laboratory Animal Resources at the University of Cincinnati Medical Center. All the animal experiments conformed to the Guidelines for the Care and Use of Laboratory Animals prepared by the National Academy of Sciences, published by the National Institutes of Health, and approved by the University of Cincinnati Animal Care and Use Committee.

### Isolation of Mesenchymal Stem Cells

Mouse mesenchymal stem cells (MSCs) are generally isolated from the tibia and femoral marrow compartments, then cultured in a medium with Dulbecco’s modified Eagle’s medium (DMEM) containing 15% of fetal bovine serum (FBS; Sigma), 2 mm L-glutamine (Gibco, USA), 100 u/ml penicillin and 100 u/ml streptomycin(Sigma), as described previously^46^. Culture medium was changed every 3–4 days, and non-adherent hematopoietic cells were removed in this process. Subsequent passages were performed with a 0.025% Trypsin containing 0.02% EDTA for 10 min at 37 °C. The third passage MSCs were used for experiments in this study. Characterization of MSCs was assayed using immuno-staining, as described previously^36^. The purified primary antibodies against mouse CD29, Sca-1, and CD34 (all diluted at 1:100) were purchased from Affymetrix-eBioscience. The Alexa Fluor® 488 Goat Anti-Rat IgG (H + L) Antibody (Life Technologies) was used as secondary antibody (dilution at 1:500).

### Isolation and Characterizations of Exosomes

One day before collection of exosomes from MSCs, medium was replaced with that containing 10% of exosome-depleted FBS (System Biosciences Inc.)]. Exosomes were isolated for *in vivo* injection and cell culture, according to the method described before^10^. Briefly, supernatants from cultured MSCs were collected on ice and centrifuged at 3,000 rpm for 30 min to remove any cells and cellular debris, and then supernatants were transferred to a fresh tube and centrifuged at 13,000 rpm for 30 min at 4°C to further remove cellular debris, shedding vesicles and apoptotic bodies. Subsequently, supernatants were filtered through 0.2 μm membrane filters (Thermo Scientific) and then centrifuged at 36,000 rpm (Ti-45 rotor) for 3 h at 4 °C. The exosomal pellet was first washed with Tris (50 mM)-buffered saline (NaCl, 150 mM) with EDTA (0.1 mM) to avoid contamination with secreted proteins^47^ and then re-suspended with incomplete DMEM medium or PBS. The quality of exosomes was confirmed by dynamic light scattering using a particle and molecular size analyzer (ZetasizerNano ZS, Malvern Instruments) according to the manufacturer’s instructions. The quantity of exosomes was determined by the Micro-BCA assay (Pierce, Rockford, IL) for measurement of total protein.

### Culture of Macrophages or Cardiomyocytes

Murine macrophage RAW264.7 cells (#91062702) were purchased from Sigma-Aldrich, and cultured in DMEM supplemented with 2 mM Glutamine, 10% FBS, 100 u/ml penicillin and 100 u/ml streptomycin. For the inflammation response assay, RAW264.7 cells were treated with LPS (100 ng/ml) for 24 h and then collected supernatants for cytokine measurements. Primary cardiomyocytes were isolated from adult rat hearts and cultured in dishes or plates pre-coated with mouse laminin (10 μg/ml), as described previously^23^. For the cardiomyocyte survival assay, the culture was treated with LPS (1 μg/ml) for 16 h, followed by addition of 20 μl MTS dye per 100 μl medium. The absorbance of the dye solution was then measured at OD490 nm, which correlates with the number of viable cardiomyocytes (CellTiter 96 AQueous One Solution Cell Proliferation Assay Kit, Promega).

### Co-Culture of MSCs with Macrophages or Cardiomyocytes

Cell co-culture was performed using a double-chamber co-culture system with a 0.4 μm pore size membrane (12-well insert, BD Biosciences) separating the upper and lower chamber cells. MSCs (1 × 10^5^ cells/well) were cultured in the upper chamber and macrophages RAW264.7 (1 × 10^5^ cells/well) were cultured in the lower chamber, followed by addition of LPS (100 ng/ml) for 12 h and then collected supernatants for cytokine assays. When MSCs were co-cultured with cardiomyocytes, adult rat cardiomyocytes were isolated and cultured in the lower chamber (1 × 10^4^ cells/well) pre-coated with laminin (10 μg/ml), as described previously^23^. For blocking of exosome release from co-cultured MSCs, these cells were pre-treated with GW-4869 (20 μM, Sigma-Aldrich) for 1 h, followed by addition of LPS for 16 h, as previously described^13^.

### Exosome Labeling

Exosomes were labelled with a PKH26 Red Fluorescent dye (Sigma-Aldrich), at a dilution of 1:200, according to the manufacturer’s instructions. Briefly, the exosomal pellet was re-suspended in 1 ml PKH26 solution and incubated for 10 minutes. After ultracentrifugation at 100,000 g for 70 min at 4 °C, the exosomal pellet was washed once in PBS, followed by centrifugation for 90 min at 150, 000 g to remove free dye. Then the pellet was re-suspended in 500 μl PBS. For *in vivo* study, red dye PKH26-labeled exosomes were injected *i.v.* into mice and PBS was used as controls; 6–8 h later, animals were sacrificed with an anesthetic overdose and hearts were isolated. The myocardium was embedded in Tissue-Tek^TM^ CRYO-OCT (Fisher Scientific) and processed for 5-μm sections. Cardiomyocytes in these sections were stained using a green dye Alexa Fluor 488 labeled α-actinin (Sigma-Aldrich). Distribution of red dye PKH26-labeled exosomes in the myocardium was examined under the LSM510 confocal fluorescence microscopy. In another set of experiment, hearts collected from exosome-injected mice and control mice were subjected to RT-PCR and Western-blotting analysis.

### Polymicrobial Sepsis Model and Treatment with MSCs or Exosomes

Polymicrobial sepsis was surgically induced by cecal ligation and puncture (CLP) as previously described^22^. Briefly, mice (8–10-week old, male, C57BL/6) were anesthetized by isoflurane inhalation and ventilated with room air using a rodent ventilator. A 1- to 2-cm midline incision was made below the diaphragm to expose the cecum. The cecum was ligated at 1.0 cm from the tip with a 5–0 sterile silk suture. A single through and through puncture was made at the middle between the ligation and the tip of the cecum with an 18-gauge to induce a severe septic injury. After puncturing, the cecum was gently squeezed to extrude a small amount of feces and returned to the abdominal cavity. The abdominal wall incision was closed in layers. After surgery, pre-warmed normal saline (0.05–0.1 ml/g body weight) was administered subcutaneously. Post-operative pain control was managed with subcutaneous injection of bupivacain and buprenorphine. Sham controls were exposed to the same surgery; however, their cecum was neither ligated nor punctured. One hour after CLP surgery, MSCs (1 × 10^6^ cells in 150 μl of PBS) or MSC-derived exosomes (2 μg/g body weight in 150 μl of incomplete culture medium) were injected intravenously through the tail or jugular vein. Same volume of PBS or incomplete culture medium was injected as controls. The survival rate was monitored every 12 h (MSC treatment) or 4h (exosome treatment) for 3 days.

### Assessment of Cardiac Function *in vivo*

Cardiac function was assessed *in vivo* using trans-thoracic M-model echocardiography (Vevo^®^2100 Imaging System, Visualsonics) with a 15-MHz probe^22^. After the induction of general anesthesia with isoflurane gas, hearts were imaged in 2-D and M-mode recorded through the anterior and posterior LV walls. Anterior and posterior wall thicknesses (end-diastolic and end-systolic) and LV internal dimensions were measured using a modification of the American Society for Echocardiography leading edge method from at least three consecutive cardiac cycles on the M-mode tracings. LV ejection fraction (EF) was calculated as: EF (%) = [left ventricular end-diastolic dimension (LVEDd)^3^—left ventricular end systolic dimension (LVESd)^3^/(LVEDd)^3^] × 100. LV fractional shortening (FS) was determined as [(LVEDd–LVESd)/LVEDd] × 100.

### Assessment of Cardiac Apoptosis *in vivo*

*In situ* cardiomyocyte apoptosis was assessed using the Dead-End Fluorometric TUNEL system (Promega), followed by staining with an anti–α-sarcomeric actin antibody (cardiomyocyte marker, Sigma-Aldrich) and 4′, 6-diamidino-2-phenylindole (DAPI, cell nucleus marker, Invitrogen)^23^. TUNEL-positive (green) nuclei were counted from 10 randomly-chosen microscope fields of the mid-ventricular section and were expressed as a percentage of total nuclei (both blue and green staining nuclei) from the same fields, using five hearts for each group and two sections for each heart. For more accurate quantification of apoptosis in sepsis hearts, DNA fragmentation was determined by a cell-death-detection ELISA kit (Roche Applied Science). Results were normalized to the standard provided in the kit and expressed as a fold increase over control.

### Cytokine Analysis in Peripheral Blood and Cell Culture Supernatants

At 12 h after CLP surgery, whole blood samples were collected by cardiac puncture, using heparinized needles, and spun down at 10000 rpm for 10 minutes to collect serum. Cell culture supernatants were harvested at 12 h after co-culturing macrophages with MSCs or with exosomes (20 μg/ml). The levels of tumor necrosis factor-*α* (TNF-*α*), interleukin 6 (IL-6) and IL-1β in the sera and culture supernatants were determined, using commercially available ELISA kits (Thermo Scientific-Pierce), according to manufacturer’s protocol. Cytokine levels were established by comparison to a standard curve per the manufacturer’s instructions.

### Quantitative RT-PCR

Total RNA was isolated from mouse hearts, MSCs, MSC-derived exosomes, or exosome-treated samples, using a miRNeasy Mini kit (Qiagen). The concentration of RNA was measured by a Nano-Drop ND-1000 Spectrophotometer (Nano-Drop Tech., Rockland, DE). The quantitative real time-PCR (qRT-PCR) for measurements of miR-223-5p and 3p was performed using a miScript PCR starter kit (#218193, Qiagen) which includes a universal reverse primer. The forward primers for real-time PCR were designed as follows: miR-223-3p forward, 5′-GCAGAGTGTCAGTTTGTCAAAT; miR-223-5p forward, 5′-GCAGAGCGTGTATTTGACAAG; miR-320 forward, 5′-GCAGAG AAAAGCTGGGT TGAG. U6 RT and reverse primer: 5′-GTGCAGGGTCCGAGGT; Forward primer: 5′-CTCGCTTCGGCAGCACA. All RT-PCR reactions, including no-template controls and RT minus controls, were run in triplicate in a CFX96 Real Time PCR system (Bio-Rad). The relative expression was calculated using the following equation: relative gene expression = 2^− (ΔCtsample−ΔCtcontrol)^.

### Western Blot Analysis

Protein samples were extracted from exosomes, cells or tissues with procedures as described in detail elsewhere^23^. Equal amounts of protein were subjected to SDS-PAGE. Binding of the primary antibody was detected by peroxidase-conjugated secondary antibodies and enhanced chemiluminescence (Amersham Pharmacia), and bands were quantified with densitometry. The source of antibodies and dilutions used were as follows: rabbit anti-CD63 (sc-15363, 1:500 dilution), rabbit anti-CD81 (sc-9158, 1:400 dilution), and a primary antibody against Sema3A (#PAB7888, dilution 1:400, Abnova Co.), or Stat3 (SC-482, dilution 1:200 Santa Cruz Biotechnology). Either α–Actin or β-Actin (1:1000 dilution, Sigma-Aldrich) was used as an internal control.

### Statistical Analysis

Data were expressed as means ± SEM. Significance was determined by Student *t* test, one- or two-way analysis of variance where appropriate to determine differences within groups. The survival rates were constructed using the Kaplan–Meier method, and differences in mortality were compared using the log-rank-test. A *P* < 0.05 was considered statistically significant.

## Additional Information

**How to cite this article**: Wang, X. *et al.* Exosomal miR-223 Contributes to Mesenchymal Stem Cell-Elicited Cardioprotection in Polymicrobial Sepsis. *Sci. Rep.* **5**, 13721; doi: 10.1038/srep13721 (2015).

## Supplementary Material

Supplementary Information
