# Supplementary material for: Exosomal miR-223 Contributes to Mesenchymal Stem Cell-Elicited Cardioprotection in Polymicrobial Sepsis
Source: Sci Rep. 2015 Sep 8;5:13721. doi: 10.1038/srep13721 (PMC4562230; doi:10.1038/srep13721)
Supplement: Supplementary Information [file srep13721-s1.pdf]

Exosomal miR-223 Contributes to Mesenchymal Stem Cell-Elicited Cardioprotection in Polymicrobial Sepsis

Xiaohong Wang[1], Haitao Gu[1], Dongze Qin[1, 4], Liwang Yang[1, 5], Wei Huang[2], Kobina Essandoh[1], Yigang Wang[2], Charles C. Caldwell[3], Tianqing Peng[6], Basilia Zingarelli[7], Guo-Chang Fan[1*]

1, Department of Pharmacology and Cell Biophysics; 2, Department of Pathology and Laboratory Medicine; 3, Department of Surgery, University of Cincinnati College of Medicine, Cincinnati, OH, USA; 4, Shanxi Medical University, Taiyuan, China; 5, Shanxi University of Traditional Chinese Medicine, Taiyuan, China; 6, Critical Illness Research, Lawson Health Research Institute, Ontario, Canada N6A 4G5; 7, Division of Critical Care Medicine, Cincinnati Children's Hospital Medical Center, Cincinnati, OH, USA.

* Correspondence to Guo-Chang Fan, PhD
Department of Pharmacology and Cell Biophysics
University of Cincinnati College of Medicine
231 Albert Sabin Way
Cincinnati, OH45267-0575
Phone: (513) 558-2340
Fax: (513) 558-2269
Email: fangg@ucmail.uc.edu

Supplementary information

Supplemental Figure S1

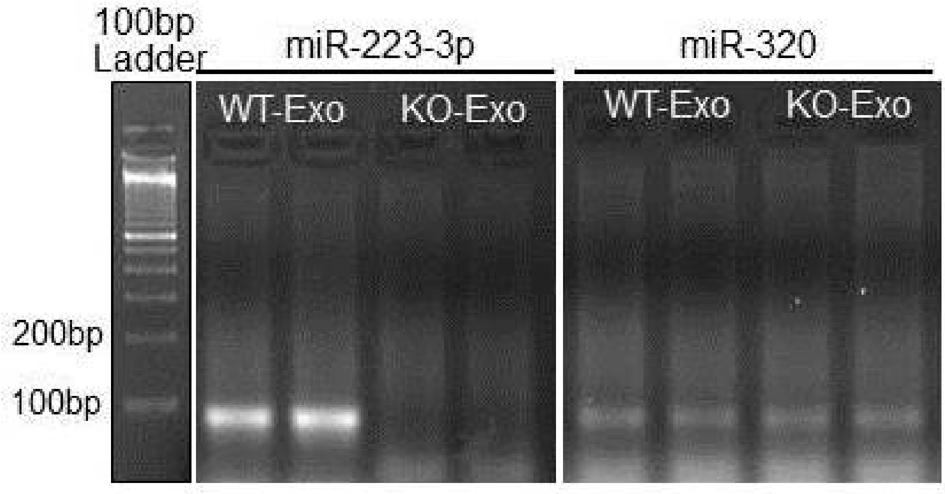

Figure S1: Exosomes were purified from culture supernatants of WT-MSCs and miR-223-KO MSCs, using sucrose gradient ultracentrifugation. Briefly, Cell culture supernatants were centrifuged for 10 min at 300g, 10min at 1200g (2X), 30min at 10000g, 60min at 70000g sequentially. The pellets were re-suspended in 5 ml of 2.6 M sucrose, 20 mM Tris-HCl, pH 7.2, and floated into an overlaid linear sucrose gradient (0.25-2.0 M sucrose, 20 mM Tris-HCl, pH 7.2) in a SW41 tube for 16 h at 100,000g. This yielded a distinct band at the density of ~1.15g/mL, as expected exosome fraction (1.13-1.19 g/mL). We collected this fraction to isolate total RNA for RT-PCR. The results of RT-PCR further confirmed that exosomes derived WT-MSCs (WT-Exo) did contain miR-223, whereas it was not encased in exosomes derived miR-223-KO MSCs (KO-Exo). MiR-320 was used as internal controls.

Supplemental Figure S2

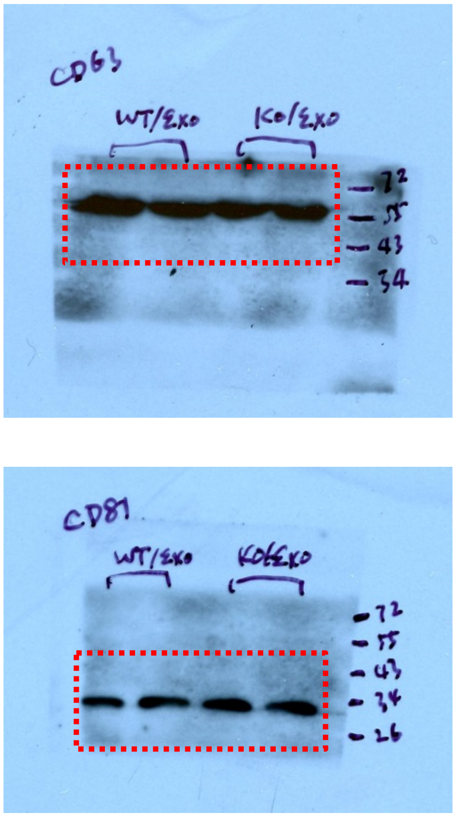

Figure S2: These original membranes were cropped for Figure 5C. The cropping lines are indicated clearly in images.

Supplemental Figure S3

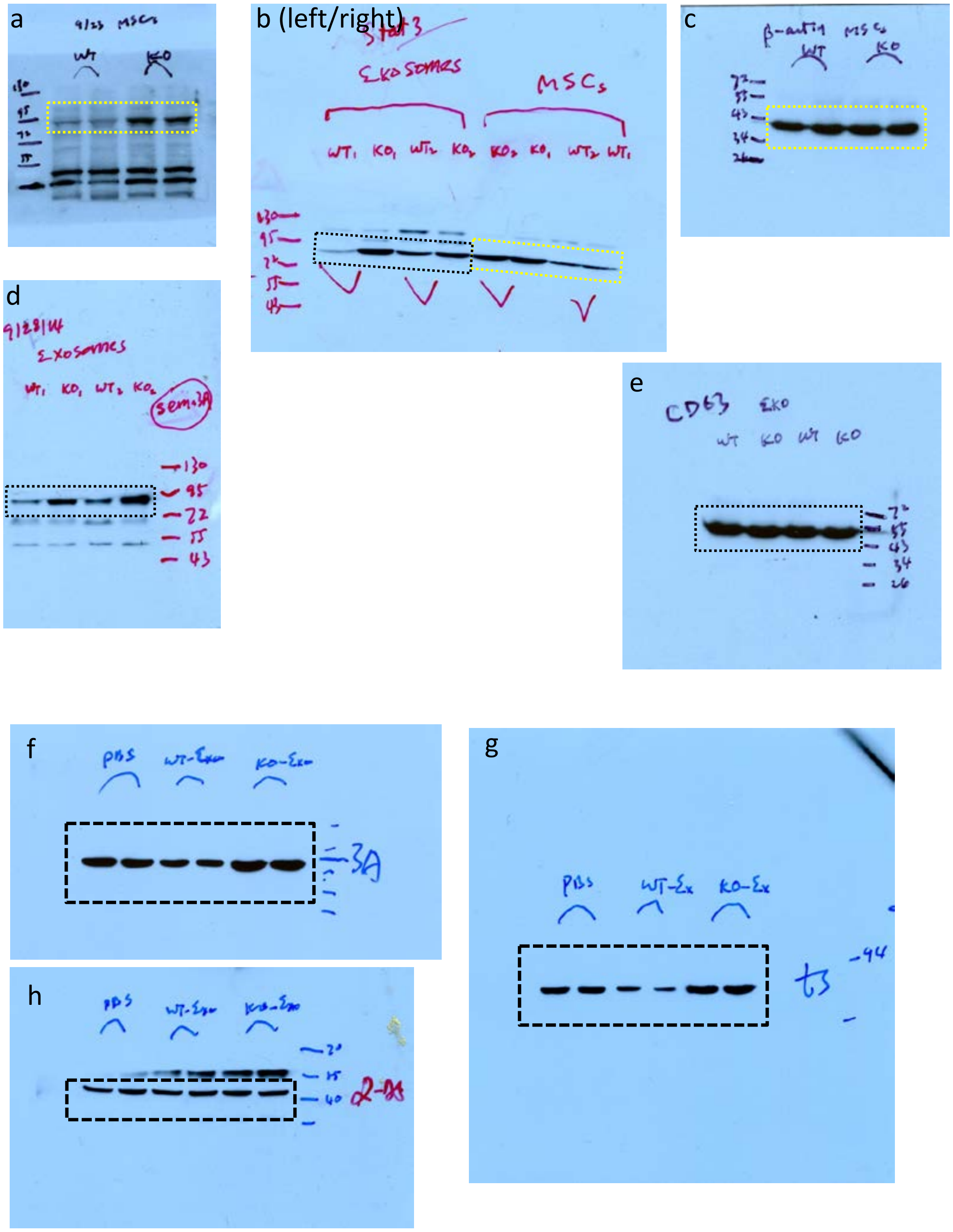

Figure S3: These original membranes a, b-right and c were cropped for Figure 7B; d, b-left and e for Figure 7D; f, g and h for Figure 7I. The cropping lines are indicated clearly in images. The molecular weight (MW) of Sema3A is predicted to be 95kDa. The MW of Stat3 is predicted to be 86kDa. The MW of actin is approximately 42kDa.
